# Levofloxacin: Insights Into Antibiotic Resistance and Product Quality

**DOI:** 10.3389/fphar.2019.00881

**Published:** 2019-08-14

**Authors:** Ensieh Izadi, Gull Afshan, Rahul P. Patel, Venkatesan M. Rao, Kai Bin Liew, Meor Mohd Redzuan Meor Mohd Affandi, Nurolaini Kifli, Amal Suleiman, Kah Seng Lee, Md. Moklesur R. Sarker, Syed Tabish Zaidi, Long Chiau Ming

**Affiliations:** ^1^Division of Pharmacy, School of Medicine, University of Tasmania, Hobart, TAS, Australia; ^2^Department of Pulmonary Medicine, Liaquat National Hospital and Medical College, Karachi, Pakistan; ^3^Faculty of Pharmacy, SEGi University, Kota Damansara, Malaysia; ^4^Faculty of Pharmacy, Cyberjaya University College of Medical Sciences, Cyberjaya, Malaysia; ^5^Faculty of Pharmacy, Universiti Teknologi MARA, Puncak Alam, Malaysia; ^6^PAPRSB Institute of Health Sciences, Universiti Brunei Darussalam, Gadong, Brunei Darussalam; ^7^College of Pharmacy, Prince Sattam Bin Abdulaziz University, Al Kharj, Saudi Arabia; ^8^Department of Pharmacy, State University of Bangladesh, Dhaka, Bangladesh; ^9^School of Healthcare, University of Leeds, Leeds, United Kingdom

**Keywords:** counterfeit, post marketing surveillance, regulatory requirement, fluroquinolone resistance, quality medicine

## Abstract

Counterfeit and substandard medicines are recognized as one of serious threats to public health. The product quality of antibacterial medicine will compromise patients’ recovery and increase the chance of antibacterial resistance. The review aims to provide a summary of low quality levofloxacin issues and the risk factors as well as suggesting the aspects of product quality that need to be regulated strictly. Quality of the active ingredient, levofloxacin, has an important role to contribute to successful therapy. The poor quality of raw material, directly and indirectly, causes treatment failure as the presence of insufficient dose, mislabeled content, and poor dissolution characteristics can lead to lower bioavailability. Identifying and reporting these factors can potentially help in improving the quality of drug marketed in various developing countries and may also reduce the incidences of treatment failure. Dissolution test is used for testing the dissolution profiles and the rate of drug release from solid formulation such as oral formulations, thus providing information regarding the in vivo performance of a formulation and its bioequivalence. On the other hand, quality-testing procedures are used for comparing the quality of products.

## Introduction

Nowadays, treatment failure of infectious diseases is one of the major healthcare issues especially in developing countries (Lee et al., 2015). Respiratory tract infection (RTI) is one of the main microbial infections in developing countries that causes about 4–5 million deaths per year among children (Liu et al., 2014). For instance, pneumonia is an inflammation of air sacs in lungs and one of common RTI that may cause hospitalization and mortality (Lee et al., 2015; Crispian, 2016). Although the emergence of antibiotic therapy and vaccines in the twentieth century increased the survival rate among these patients, however, it was still reported that pneumonia caused a high incidence of death rate among geriatrics and children especially in the developing countries (Basnet et al., 2015). There are resistance cases reported on first-line antibiotics such as penicillin and macrolides for the treatment of pneumonia. Quinolone class is considered as one of the empirical treatments of the infection (Endimiani et al., 2005). However, the rising of quinolone resistance cases is alarming in North and South American countries in the year 2000 (Kays et al., 2002). *Haemophilus influenzae* is one of common organisms which can cause RTI, and there are few studies reported the quinolone-resistant of *H. influenzae* (Bastida et al., 2003). There are few studies suggesting that poor quality and substandard drugs are a major contributor to antibiotic resistance in RTIs in developing countries (Conway et al., 2013; Currie et al., 2014; Basnet et al., 2015). This draws the attention of regulatory authority to look into the aspect of poor quality medicines which were reported in many developing countries. It is reported that a significant percentage of generic drugs fails to meet the innovator specifications in Latin America (Nightingale, 2005).

Drug quality has a key role to deliver successful treatments. It is important to achieve the required minimum inhibitory concentration (MIC) in order to cure an infection (Miller et al., 2005). High quality antibiotics are essential in treatment of serious common RTIs while poor quality antibiotic therapy may increase morbidity and mortality rates (Khasawneh et al., 2014). Hence, the aim of this review is to summarize low quality levofloxacin tablet issue and suggest aspects of product quality that need to be strengthen and regulated strictly.

## Levofloxacin Resistance

Previous studies conducted in South Korea, Canada, USA, and Spain show the correlation between fluoroquinolone administration and resistance (Kays et al., 2002). The resistance of microbes toward some fluoroquinolones products have been reported in the treatment of hospital acquired infections (Torloni et al., 2016). Kays et al. (2002) have claimed that levofloxacin susceptibility in pneumonia infection had reduced as utilization of levofloxacin prescription increased. Meanwhile, Davidson et al. (2002) reported treatment failure in *Streptococcus pneumoniae* in three different cases after fluoroquinolone administration. Fluoroquinolone resistance is reported in some developing countries such as China and Thailand with 3% non-susceptibility to levofloxacin while the fluoroquinolone resistance against *S. pneumonia* is below 1% in the United States from 1987 to 1998 (Sahm et al., 2000; Hu et al., 2011). Although, the results in the United States may be related to different prescribing behaviors or fundamental characteristics of the selected pathogens, but *S. pneumonia* is still capable of resistance development in fluoroquinolone therapy (Sahm et al., 2000; Davidson et al., 2002). Seok et al. (2018) have stated evidence for non-susceptibility to levofloxacin due to prior usage of antibiotics within 3 months, and it is recommended to restrict unnecessary fluoroquinolone prescriptions. Also, Ayukekbong et al. (2017) have presented multiple factors contributing to the problem of antimicrobial resistance due to unnecessary antibiotic prescriptions, especially in developing countries. Available evidence suggests that non-susceptibility to levofloxacin is an ongoing problem.

## Poor Quality Drug Products

Poor quality drug product is a major global healthcare issues (Sarpong and Miller, 2014). There are studies suggesting that antibacterial drugs mainly antimalarial, antiviral, and antibiotics are among the poor quality drug products reported in developing countries (Nsimba, 2008; Melo et al., 2014). Poor quality drug product is defined as drug product which the drug content does not follow the provided standards such as chemical stability, bioavailability, and purity in drugs (Almuzaini et al., 2013). Poor quality drug product can be sub-classified into substandard drugs product and counterfeit drug product (Newton et al., 2010). Substandard drug products are medicines produced by an authorized manufacturer which do not follow designated standard quality standings. On the other hand, counterfeit medicines are defined as “deliberately and fraudulently mislabeled with respect to identity and/or source” whether with the right or falsified ingredients or with the forged labeling. Substandard/counterfeit medicines are not usually bioequivalent due to differences in bioavailability as a result of pharmacokinetics (Johansson et al., 2010).

Moreover, some poor quality drugs do not have good *in vivo* dissolution in the gastrointestinal tract leading to incomplete absorption; hence, therapeutic levels in blood cannot be achieved (Johansson et al., 2010). The MIC is not achieved when the drug plasma concentration is below expectation which leads to therapy failure and antibiotic resistance (Almuzaini et al., 2013).

**Table 1** shows the relevant studies which reported the substandard/counterfeit drugs. These studies claim that there are many substandard or counterfeit drugs available in developing counties (Nightingale, 2005; Hosseini et al., 2011; Bate et al., 2013; Khurelbat et al., 2014; Lalani et al., 2015).

**Table 1:** The percentage of substandard, counterfeit, and unregistered levofloxacin available in the market of different countries.

Regions	Substandard/counterfeit (%)	Total samples	References
Africa India Middle income countries	16.6 10.1 3.9	713 *	(Bate et al., 2013)
Iran	9.1	7910	(Hosseini et al., 2011)
Mongolia	18	388	(Khurelbat et al., 2014)
Afghanistan	26	126	(Lalani et al., 2015)
13 countries including Asia and Latin America	20	40	(Nightingale, 2005)

*The studies were conducted in different countries such as Angola, Brazil, China, Democratic Republic of Congo, Egypt, Ethiopia, Ghana, India, Kenya, Nigeria, Russia, Rwanda, Thailand, Turkey, Uganda, and United Republics of Tanzania and Zambia.

In addition, many studies have confirmed that substandard or counterfeit drugs caused side effects such as allergies because of impurity, toxicity, or a high percentage of the active agent (Tipke et al., 2008; Wang et al., 2015). Substandard drugs may lead to potential side effects and death as the higher or lower amount of drug than the labeled one is delivered (Boyd et al., 2009; Tamargo et al., 2015; Wang et al., 2015).

## Aspects of Product Quality

It is important that the generic pharmaceutical manufacturers ensure the production of quality products which help to reduce the therapy failure and antibiotic resistance. Several aspects of product quality is discussed in this review—namely, dissolution study and bioequivalence, quality control of product, quality control test and stability of drug product, post-marketing surveillance (PMS), effect of storage, and poor quality control during manufacturing.

## Dissolution Study and Bioequivalence

A generic drug is defined as a product which has the same active ingredients as the innovator medicine but with different inactive agents or excipients (Nitzki-George, 2004). Generic drug products are cheaper than the innovator drug products due to the initial saving of research and development expenses for new drugs, so they are highly preferred in the society especially by low income patients (Dunne et al., 2013).

One of the ways to show drug quality of generic product is through bioequivalence study. Bioequivalence study is required to ensure that the generic medicine product is equivalent with the innovator medicine product in terms of pharmacokinetics requirement as maximal serum concentration and area under the curve (drug concentration against time) (Johansson et al., 2010). Hence, FDA uses a bioequivalent approach to assess generic drugs (Paveliu et al., 2011). A bioequivalent is considered if the two drugs in comparison have less than 20% difference in the pharmacokinetics parameters (Paveliu et al., 2011). Thus, generic drug manufacturers are required to prove that rate and extent of drug absorption into the bloodstream is comparable to the amount from a brand name product (Davit et al., 2009).

Although, both generic and innovator drugs are susceptible to be falsified, but generic medications are easier to be manipulated (Gibson, 2005). In most cases, samples of generic drugs are as safe and effective as branded one, but still some problems may occur (Kesselheim et al., 2008). Treatment failures have been reported in different cases, such that in one case, symptoms were improved after replacing the prescribed generic levofloxacin with Tavanic (Innovator product) without the presence of any adverse effect (Gallelli et al., 2013). The issue was identified as the excipients of the generic product is different (Tamargo et al., 2015). Although the active content that helps to control may be the same, the filler material, color, or binder used may be different (Tamargo et al., 2015). The problem may occur when the active agent is not sufficient or the inactive content has impurity or contamination that can have a great impact on bioavailability, metabolism, and elimination (Tamargo et al., 2015).

## Quality Control and Stability of Drug Product

Pharmaceutical industries need to adhere and comply with the good manufacturing practice (GMP) to guarantee the products safety, efficacy, and quality (Li et al., 2015). Nowadays, export or occasional drug use among the country of production is also subjected to compliance with GMP requirements and regulations by manufacturer (Abdellah et al., 2015). Compliance with the requirements and regulation of GMP is considered as an essential and vital aim for quality assurance and ultimately the profitability of the pharmaceutical industry (Tomic et al., 2010).

Analysis of raw materials, substances, and intermediate products are important tasks in pharmaceutical manufacturing and quality control. Pharmacopeia is used as a reference for chemical, physical, and microbiological tests (Kaul et al., 2016). The manufacturers are responsible to not just produce a final product, but they are also obliged to look into the post-production drug product stability issues (Lambert and Conway, 2003; Wang et al., 2015; Kaul et al., 2016). U.S. Pharmacopeia (USP) is one of commonly available pharmacopeia to control quality of raw materials, components, packaging, and final products (Panchagnula et al., 2006; Kaul et al., 2016).

Innovator (brand name) and generic medicines use the same API, but they may contain diverse inactive agents while they still need to follow quality standards of innovator drug (Al Ameri et al., 2012). Quality control is essential for detecting the substandard drugs containing inactive substance, impurities, and toxic substances (Almuzaini et al., 2013; Fadeyi et al., 2015). Substandard drugs can be detected using the quality-testing protocols such as USP which is also defined by the World Health Organization and International Pharmacopeias (Kayumba et al., 2004; Maezawa et al., 2013).

The drug product should contain an equal amount of active pharmaceutical ingredient (API) as stated in the product claim (Zaid et al., 2013). According to WHO’s definition, an API is a material or combination of materials that are used in the pharmaceutical products as an active compound with a direct effect in treatment (World Health Organization, 2011; World Health Organization, 2016). The compatibility between API and excipients is important to ensure stability of the API in the dosage form. A stable API will deliver the claimed effect within the shelf life of the product. A crucial role of excipient in drug formulation requires the regulatory approval to avoid toxic or poor quality medicines that may lead to treatment failure (Baldrick, 2000).

### Effect of Storage Conditions on Drug Stability

Storage is one factor that may affect the quality of a drug (Kayumba et al., 2004). The storage under tropical conditions was reported by Kayumba et al. (2004) as the main problem of the instability of some formulations of drugs collected in Tanzania, and it was suggested to drug regulatory authorities to develop a quality assurance system to control poor quality drugs. Another study by Nogueira et al. (2011) claims that storage has an essential role in the stability of the drug after production, and poor drug samples were found in poor drug storage conditions in 75% relative humidity and 40°C.

### Poor Quality Control During Manufacturing

Quality control framework based on established pharmacopeia techniques is essential to ensure product quality (Losada-Urzáiz et al., 2015). Moreover, lack of quality control due to irresponsibility and corruption increase the percentage of supplying counterfeit drugs in the market which lead to serious public health issues (Zavras et al., 2016). One of the main reasons for quality problems in developing or low income countries is lack of PMS and regulation (Taylor et al., 2001). The financial issue may increase the percentage of treatment failure and resistance due to poor quality or counterfeit drugs in developing countries (Taylor et al., 2001; Nsimba, 2008). Nevertheless, the number of cases of substandard or poor quality drugs are significant in low income and developing countries which can be related to limited financial support in health care, PMS, and pharmaceutical manufacturing (Taylor et al., 2001; Tipke et al., 2008; Conway et al., 2013; Karunamoorthi, 2014; Fadeyi et al., 2015).

### Control Measures to Reduce Low Quality Drugs

Low quality medicine produced during pharmaceutical manufacturing can be detected using drug dissolution studies (Wang et al., 2015). The most effective way to reduce the low quality drugs in the market is strictly prohibiting entry of counterfeit drugs into the market (Losada-Urzáiz et al., 2015). The other way to ensure both innovator and generic drugs are following standards and regulations is clearly expressed to the manufacturers that the specific pharmacopoeial techniques need to be complied (Losada-Urzáiz et al., 2015). Bioequivalence study is a must to ensure that the generic product is equivalent with the innovator product.

## Case Study: Levofloxacin

Levofloxacin is a broad spectrum antibiotic which belongs to the third generation of quinolones group (Scoper, 2008). It prevents DNA replication in bacteria leading to bacterial cell death, and it is mainly used as a second-line therapy of lungs infection (Noreddin and Elkhatib, 2010). This drug is considered as an important antibiotic in case of microorganism that is resistant to other class of antibacterial agents such as beta-lactam antibiotics; hence, its quality is critical in effective treatment (Kayumba et al., 2004; Endimiani et al., 2005).

Levofloxacin is chosen as the model drug for a common empirical treatment of pneumonia caused by Gram-negative bacteria such as *H. influenza* as well as Gram-positive bacteria such as *S. pneumoniae* (Kays et al., 2002).

Levofloxacin has high oral bioavailability with rapid bactericidal activity; hence, it is considered for the treatment of choice for severe pneumonia (Boyd et al., 2009; Maezawa et al., 2013). Although, levofloxacin is one of the most widely prescribed agents for the pneumonia treatment but the number of treatment failure incidences have been recently increased with levofloxacin therapy (Kays et al., 2002; Bastida et al., 2003; Endimiani et al., 2005; Boyd et al., 2009). The possible risk factors suggested for the treatment failure of levofloxacin are poor quality medicines and drug resistance (Davidson et al., 2002). Death in pneumonia due to levofloxacin treatment failure has been reported in North America as four patients in Canada and one patient in the USA in 2000–2001 (Davidson et al., 2002; Kays et al., 2002). Pneumonia can be caused by virus, bacteria, or fungi, and it is commonly categorized as community-acquired pneumonia (CAP) and hospital-acquired pneumonia (HAP) (Abrahamian et al., 2008). The CAP is caused by *Streptococcus pneumoniae, Haemophilus influenzae, Chlamydia pneumoniae*, and respiratory viruses (Abrahamian et al., 2008; Johansson et al., 2010). On the other hand, HAP occurs after 48 h of hospital admission by *Escherichia coli, Klebsiella pneumoniae, Pseudomonas aeruginosa*, and *Staphylococcus aureus*, including methicillin-resistant *S. aureus* (MRSA) (Imran et al., 2016). The treatment is chosen on the basis of different categories and the causative organisms (Imran et al., 2016). The main treatment of pneumonia is antibiotics while sometimes the patients need to be admitted to the hospital to monitor their progression (Bradley et al., 2014). In addition, approving a specific microbial etiology is important in empirical treatment of pneumonia to minimize the risk of exposed side effects and antibiotic resistance (Leekha et al., 2011). There are different types of antibiotics used for treatment of pneumonia especially in CAP which is the most common pneumonia and may cause serious infections globally (Abrahamian et al., 2008). Although, the particular antibiotic for management of CAP is given based on severity of infections or etiology (pathology) while combination of beta-lactam (amoxicillin) and macrolides (azithromycin) or antipneumococcal fluoroquinolone are the preferred empirical treatment (Mandell et al., 2007).

Therapeutic window is the range between the amount of the drug that gives the desired effect and the drug may be ineffective or toxic if the drug therapeutic index is below or above the range, respectively (Tamargo et al., 2015). Treatment failure may occur by various factors such as an inadequate antibiotic coverage and concentration, deficient diagnosis, or resistance (Sánchez García, 2009). Poor quality antibacterial may lead to drug resistance or severe side effects if the therapeutic window of levofloxacin is not achieved (Boyd et al., 2009; Tamargo et al., 2015). Therefore, if the dose is lower than the standard window, the drug is not effective, and it may increase resistance; on the other hand, if above the MIC range, then it may cause toxicity and adverse reaction (Tamargo et al., 2015). Additionally, levofloxacin is a broad spectrum antibiotic which is susceptible to cause resistance of normal flora; hence, substandard drugs may increase the risk of resistance significantly (Kays et al., 2002; Bastida et al., 2003).

Poor quality antibacterial product may lead to drug resistance, severe side effects, and death; hence, it has a critical role in the treatment and public health (Melo et al., 2014). There are many factors causing resistance, such as irrational and excessive use caused by wrong prescribing and poor quality of medicine (Melo et al., 2014). **Table 2** shows the relevant studies reported levofloxacin resistance to *H. influenza, S. pneumoniae*, and *S. aureus* species in different pneumonia. Even though, the site of infections and treatments are different from each other, but it shows levofloxacin resistance have occurred (Ho et al., 2001; Davidson et al., 2002; Kays et al., 2002; Bastida et al., 2003; Endimiani et al., 2005). Thus, it is evident that levofloxacin resistance is one of the main reasons behind treatment failure of pneumonia (Endimiani et al., 2005). Therefore, public awareness about low quality medicine can be created through the reported resistant susceptibility and quality of levofloxacin (Talan et al., 2016).

**Table 2:** Features of treatment failure of pneumonia due to levofloxacin-resistant in different countries.

Disease-causing bacteria	Reported country	References
*Haemophilus influenzae*	Spain	(Bastida et al., 2003)
*Staphylococcus aureus*	Pakistan	(Rizvi et al., 2014)
*Streptococcus pneumoniae*	Canada	(Davidson et al., 2002)
	Italy	(Endimiani et al., 2005)
	USA	(Kays et al., 2002)
	Hong Kong	(Ho et al., 2001)

### Dissolution Testing of Levofloxacin Tablets

Quality control tests used to identify substandard drugs and control the quality of drugs are colorimetric, dissolution, chromatography, and spectrometry procedures (Almuzaini et al., 2013; Maezawa et al., 2013). Dissolution test provides the data of *in vitro* performance of the drug and prediction for its bioequivalence (Anand et al., 2011). Hence, it is used to estimate the concentration of API and also drug quality (Anand et al., 2011). The dissolution rate consists of drug release by dissolving of solid form in an API into the fluid to determine the stability and physical changes (Kayumba et al., 2004; Nogueira et al., 2011). Drug dissolution test is an essential factor to identify the quality of drugs especially in oral administration as it provides the process of drug release from its solid formulation, and it can discriminate the substandard and low quality products (Lambert and Conway, 2003; Al Ameri et al., 2012; Khadka et al., 2014).

Dissolution test is required to be applied under *in vitro* conditions to compare the drug release of active ingredient from a solid form into solution and its bioavailability during a defined time (Frick et al., 1998). Previous studies on dissolution of levofloxacin have shown little variation in levofloxacin tablets tested in different countries. **Table 3** shows the various dissolution studies of levofloxacin tablets conducted in various countries.

**Table 3:** Various dissolution studies of levofloxacin tablets conducted in various countries.

Studied country	Dissolution test setting [speed (rpm)/media volume (ml)]	Samples tested (numbers)	Difference in dissolution profiles	Authors
Taiwan	50/900	4	Yes	(Sun et al., 2016)
Japan	50/900	24	Yes	(Maezawa et al., 2013)
Pakistan	50/900	6	No	(Bano et al., 2011)
Pakistan	75/1,000	3	Yes	(Nazir et al., 2013)
India	100/900	3	Yes	(Bhavanam et al., 2011)

It is observed that certain generic brands in Japan, Taiwan, Pakistan, and India showed difference in dissolution profile when compared with the innovator product. There is only one study by Bano et al. (2011) comparing few generic brands of levofloxacin tablet does not show significant difference in dissolution profile. The result of this study shows that the different *in vitro* activity may have a direct effect on the *in vivo* activities and demonstrate the predictive capability of the former (Sun et al., 2016).

## Conclusions

Current evidences from various researchers have indicated that poor quality of levofloxacin tablets could be an important factor causing resistance of levofloxacin. Clinical efficacy of this broad spectrum antibiotic relies on the quality of the levofloxacin tablet in term of in vivo release and dissolution inside the gastrointestinal tract. As poor performances of quality control tests of levofloxacin tablets have been reported in developing countries, more PMS to ensure quality of levofloxacin tablets are required to prevent therapy failure and antibiotic resistance.

## Author Contributions

RP, SZ and LM conceived the concept; EI, VMR, RP, MS, SZ and LM wrote the initial draft; KBL, AS, NK MM, KSL and GA revised the paper. All authors contributed toward finalizing the manuscript and agreed to be accountable for all aspects of the work.

## Conflict of Interest Statement

The authors declare that the research was conducted in the absence of any commercial or financial relationships that could be construed as a potential conflict of interest.
